# The human life history is adapted to exploit the adaptive advantages of culture

**DOI:** 10.1098/rstb.2019.0498

**Published:** 2020-06-01

**Authors:** Peter J. Richerson, Robert Boyd

**Affiliations:** 1 Department of Environmental Science and Policy, University of California—Davis, One Shields Avenue, Davis, CA 95616, USA; 2 School of Human Evolution and Social Change, Arizona State University, Tempe AZ, USA

**Keywords:** evolution, culture, adaptation, life-history theory

## Abstract

Humans evolved from an ape ancestor that was highly intelligent, moderately social and moderately dependent on cultural adaptations for subsistence technology (tools). By the late Pleistocene, humans had become highly dependent on culture for subsistence and for rules to organize a complex social life. Adaptation by cultural traditions transformed our life history, leading to an extended juvenile period to learn subsistence and social skills, post-reproductive survival to help conserve and transmit skills, a dependence on social support for mothers of large-brained, very dependent and nutrient-demanding offspring, males devoting substantial effort to provisioning rather than mating, and the cultivation of large social networks to tap pools in information unavailable to less social species. One measure of the success of the exploitation of culture is that the minimum inter-birth interval of humans is nearly half that of our ape relatives. Another measure is the wide geographical distribution of humans compared with other apes, based on subsistence systems adapted to fine-scale spatial environmental variation. An important macro-evolutionary question is why our big-brained, culture-intensive life-history strategy evolved so recently and in only our lineage. We suggest that increasing spatial and temporal variation in the Pleistocene favoured cultural adaptations.

This article is part of the theme issue ‘Life history and learning: how childhood, caregiving and old age shape cognition and culture in humans and other animals'.

## 1. Introduction

The basics of life-history analysis are reviewed in this issue by Nettle & Frankenhuis [1]. Several distinctive features of the human life history were described in Kaplan *et al*.'s [2] classic paper. They pointed to four characteristics of the human life history: an exceptionally long lifespan, an extended period of juvenile dependence, support of reproduction by non-reproductive individuals, especially post-reproductive individuals, and large male contributions to the support of women and children. In spite of an otherwise slow life history, human inter-birth intervals are shorter than those of chimpanzees. At the same time, adult death rates in human hunter–gatherers are rather lower than in chimpanzees [3]. Thus, the human life history leads to high potential rates of population increase compared with chimpanzees. The first two characteristics make use of our very large brain via investment in learning during the extended juvenile period and the exploitation of that investment during the long adult phase. The latter two support humans' acquisition of very large brains, which in turn are responsible for our great capacities for learning, cognition and insight according to Kaplan *et al*. [2] The other great apes and other large-brained animals (relative to their body size), such as capuchin monkeys, the toothed whales, elephants, and crow and parrot family birds, have aspects in common with the human life history, if in less extreme form. Many other species have long life histories but not large brains (e.g. Galapagos tortoises). The general issue of long life histories is too complex to be reviewed here [4]. Kaplan *et al*. [2] provide data from the South American Ache and Hiwi and the African Hadza hunter–gatherers, in comparison with chimpanzees, to make their assertions concrete. They also construct a life-history model that supports the idea that the four characteristics of humans will coevolve to yield the observed patterns. For example, a late age of maturation selects for low adult mortality to amortize the investment in a long juvenile period. See also [5–8]. Walker *et al*. [9] note that return rates to Ache hunters peak rather later than measures of strength, and ethnographic information suggests that the long time needed to acquire the necessary skills to hunt effectively accounts for the lag. Koster *et al*. [10] have recently replicated Kaplan *et al*.'s [2] work on a sample of hunters from 40 societies showing similar patterns, though with appreciable cross-cultural variation. Hill *et al*. [11] argue that the essential elements of the human complex cultural adaptation, such as our use of tools to exploit hunted and extracted resources, were already present in rudimentary form deep in the history of our lineage.

The reason that our large brain features so prominently in discussions of the evolution of the human life history is that it generates strong life-history trade-offs. Aiello & Wheeler [12] noted that nervous tissue is energetically costly per unit weight and proposed that, in humans at least, the cost of a large brain was met by shortening the gut, also a an energetically costly tissue. Our short gut means that human must exploit foods that are nutrient dense and employ food processing techniques like cooking and fermentation to increase nutrient density [13].

Isler & Van Schaik [14] argue that every species has a ‘grey ceiling,' a brain size at which the reproductive rate would fall so low that populations would be subject to the risk of extinction. Generally speaking, they argue that birds tend to have higher grey ceilings than mammals because male contributions to the raising of young are substantial in many bird species but less common in mammals. The other great apes are typical mammals. Males provide no parental care and devote virtually all their resources to mating effort. Nor do other females provide significant assistance to mothers in other apes. Human mothers by contrast have extensive allomaternal assistance [15,16]. Adults of both sexes are also cared for when they are incapacitated [3]. These authors stress the social-cognitive effects of allomaternal care in human evolution. If allomaternal care were primitive in the hominin lineage, it might have served as a preadaptation for other kinds of human cooperation. Perhaps early allomaternal care was an adaptation to the exploitation of dry environments by bipedal hominins in the late Pliocene or early Pleistocene because female foraging away from water would have been a burden on mothers' water balance if they nursed during such excursions (Lesley Newson 2016, personal communication). Kaplan *et al*. [2] suggest that a shift to the hunting and extractive niche originated our dependence on intelligence and cooperation. Alternatively, the evolution of allomaternal behaviour may have been driven directly by increasing brain size and shortening guts, which subsequently led us to exploit the hunting and extractive foraging niche, requiring bigger brains, culture and cooperation, including cooperative breeding. Human brain size increased fairly steadily during the Pleistocene, perhaps driven by increasing environmental variability [17]. The only issue at stake here seems to be whether the external force of increased climate variability played a key role. There is widespread agreement that the invasion of the hunting/extractive niche, leading to selection for larger brains and the need for cooperative breeding to provision such brains, was a key early part of our origins. Given the poor quality of the palaeoanthropological record, sorting out the precise sequence of events is difficult.

There is some controversy over who were the most important providers of alloparental care over the course of human evolution. Kristen Hawkes *et al*. [18] noted that Hadza men garnered high returns from big game hunting even though hunting small game would result in much more frequent success. Big game is shared with the hunter's band, meaning that a band comprising several good hunters would ensure a reasonably steady supply of meat and fat. This set up a public goods problem that Hawkes *et al*. believed hunter–gatherers could not solve. Hawkes [19] proposed that male hunting was a form of sexually selected ‘showing off' and that the large quantities of meat produced were evolutionarily incidental.

Hawkes *et al*. [20] went on to suggest that post-menopausal grandmothers were the most important allomaternal providers on the evidence the Hadza grandmothers produce a fair amount of calories in the form of starchy tubers. Kaplan *et al*.'s [2] data are inconsistent with this hypothesis in part because Hadza grandmothers' (and men's) production of calories is implausibly high. In the Ache and the Hiwi, grandmothers' surplus production is quite low compared with adult men's. Of course, grandmothers and other females without dependent offspring might have been the dominant allomaternal providers earlier in human evolution when a smaller brain made less demands on allomaternal provisioning. In some contemporary matrilineal societies, often tropical forest horticulturalists, women do the bulk of agricultural work and men are free to invest more heavily in showing off without compromising the viability of their offspring. Mattison *et al*. [21] label this the ‘expendable male hypothesis' and note that it is a common life-history pattern in mammals generally. It is probably misleading to talk about *the* human life history in the face of likely large changes over the Pleistocene and considerable ecological and cultural variation around the current central tendency. Another extreme human life-history variant is modern ‘demographic transition' societies that have far fewer children than are economically feasible [22]. Modernity generates small but appreciable selection on life-history traits in contemporary societies [23].

One constraint on the small game hypothesis is that human physiology precludes getting a large share of calories from lean protein. Humans cannot process enough nitrogenous waste from using protein as a source of calories above about 35% of total caloric needs, and small animals are generally very low in fat [24]. Kaplan *et al*.'s [2] data suggest that even tropical hunter–gathers often get around 50% of their calories from game (*contra* [25] but consistent with [24]). Large game is much fatter than small game. Of course, men's motives themselves are a proximal driver of behaviour, and natural selection at the band level [26] might co-opt sexual selection to produce a group benefit.

Wood & Hill [27] tested the show-off hypothesis by asking Ache hunters whether they would elect to join a band of hunters with an opportunity to distinguish themselves above other hunters, or a band with other good hunters who would supply considerably more meat to them and their families via cooperation. Men with dependent offspring overwhelmingly chose the hypothetical band with other good hunters whereas single men mostly chose the band with the greatest show-off opportunity. The men in the experiment verbalized their choices in the terms the experiment intended. Men seem to change their motives when they have dependents.

## 2. What are big brains for?

Given that our large brain coevolved with our slow life history and our cooperative breeding adaptation, it is of interest what the brain is adapted to do. Some of the controversy involves whether big brains are for managing our social life [28,29] or for adapting to subsistence and environmental challenges [30]. We view this debate as somewhat misbegotten. As Steward [31] pointed out long ago, human subsistence is gained by social means. Accordingly, human societies vary as a function of ecology and social life. The division of labour between men and women in hunting and gathering economies, compared with the comparative lack of male participation in rearing juveniles in some matrilineal societies, is an instance of this.

A different controversy involves the roles of culture and individual intelligence in the evolution of our large brain. This is a subtle question. The subtlety is twofold. First, empirically, the comparative biology of brain size suggests that it is correlated with both individual and social learning across a wide range of behaviours and species [32–34]. Reader *et al*. [35] argue that intelligence is a general cognitive capacity, a suggestion that is consistent with some recent cognitive neuroscience models of brain function [36,37]. It is as if individual and social learning share many more basic cognitive resources, for example the capacity for associative learning.

Second, Boyd & Richerson [38] showed that individual learning is an important force in cultural evolution. It acts as a non-random source of primary variation that is important at the initial stages of the spread of an innovation. The strength of selective social learning is proportional to the number of variants individuals get to compare. When a new desirable innovation is rare, this force is very weak, but the role of individual learning is maximal. In a recently changed environment, many individuals may use individual learning/creativity to adapt relatively rapidly, giving rise to variation that selective adoption can work to increase in frequency. Thus, individual learning and social learning are likely to be complementary processes. Individuals might have genetically or culturally transmitted learning strategies that mix and match the two [39,40].

Some prominent evolutionary psychologists deny that culture, in the sense of socially transmitted traditions, plays any significant part in the evolution of humans or other animals (e.g. [41]). Tooby & Cosmides [42] originally proposed that human Pleistocene adaptations evolved in the form of genetically coded specialized, encapsulated modules of which we might have hundreds or thousands. They seemed to dismiss entirely the ‘Standard Social Science Model' in which culture played a dominant role. Recent work in cognitive neuroscience casts doubt on the innate modularity hypothesis and favours large roles for individual and social learning in constructing functional cognitive circuits [36,37]. Genes seem to play a larger role in the highly conserved emotional circuitry of the brain [43], but even here the case for cultural modulation of the emotions is strong [44].

More recently, Cosmides & Tooby [45] have proposed that humans also have a powerful ‘improvisational intelligence' which allows individuals to solve complex challenges on their own (see also [46]). In these papers, the authors do not seem to doubt that culture exists and culture creates local traditions. Thus, it is not clear how to take their rejection of the Standard Social Science Model, but it is clear that they wish all the ultimate explanations to rest on the genetic evolution of the human brain. This modern human nature argument was well articulated by E. O. Wilson [47], although a heavy emphasis on genes leads to the conclusion that local genetic differences should readily evolve [48]. Tooby & Cosmides [42] insisted that no such differences exist because at equilibrium in the Pleistocene, there would be no variation in genetic traits related to fitness. This was a general claim of R. A. Fisher's that has not stood the test of time in evolutionary biology [49]. Lumsden & Wilson [48] also do not doubt that culture plays some sort of role in human evolution. One way to make sense of these authors is to assume that they are strongly influenced by the Modern Synthesis in which the only things that can truly evolve are genes [50]. Culture is fine so long as it is not taken to play other than a proximal role in human evolution.

Cultural evolutionists have proposed that culture in humans is an evolutionarily active system that can even act as a selective force on genes. Defenders of the Modern Synthesis can be quite intemperate in their rejection of such heresy [46,51]. However, there are good examples of culture-led gene–culture coevolution in the case of humans [52–54]. For example, Richerson & Boyd's [55] tribal social instincts hypothesis conjectures that group selection on *cultural* variation operating via social selection (selective rewards and punishments, [56]) acting on genes shaped our innate social psychology, making us more docile for example. If so, cultural evolution is playing an ultimate role alongside genes in human evolution. Likewise, we think that the human life history coevolved with culture, often driven by cultural innovations [57].

Cultural evolutionists argue cultural traditions like technology and social organization typically evolve cumulatively over prolonged periods, often resulting in quite sophisticated adaptations [53,55,58–60]. Boyd & Richerson [38] is an extended attempt at an evolutionary-functional analysis of social learning and human culture. In this picture, individual-level intelligence in the form of learning, creativity and selective imitation and teaching plays a key role in the evolution of sophisticated cultural traditions as cultural evolutionary forces. Relatively weak individual intelligence applied by a population of people to inventing and selecting ideas and practices generation after generation can relatively rapidly generate adaptations far more sophisticated than any single genius could invent. Even very simple artefacts like paper clips and dinner forks evolved over a period of time, involving a succession of suboptimal variants until a dominant variant emerged [61]. Some adaptive cultural practices have to be maintained in the face of individual intelligence. Henrich [53] gives the example of the detoxification of bitter manioc, which contains cyanide. Extensive leaching and toasting can render bitter manioc edible, but the long-term effects of quite small amounts of cyanide are serious, amounts below the level of detection by taste.

In essence, the cultural niche hypothesis defended by Boyd *et al*. [60] holds that the high economic and demographic productivity of adult men and women observed in contemporary foragers is made possible by cumulative culture. Kaplan *et al*. [2] point out that that, compared with chimpanzees, humans specialize in resources that require high skills to exploit. These skills are only partially mastered during the juvenile period, leading Hill *et al*. [11] to argue that most of the skills involved depend upon ecological knowledge, a sophisticated toolkit and institutionalized, cooperative social systems. Among hunter–gatherers these things delivered large amounts of meat, fat and carbohydrate-rich plant foods, shelters and clothing adequate for even extreme environments, and boats to exploit aquatic environments. All of these attributes were specialized to local variations in resources available. By at least 2.1 Ma, our genus had spread out of Africa to as far as East Asia [62], and by the late Pleistocene, people pioneered cold temperate and even Arctic environments. Likely, the ability to use culture to evolve adaptations to local environments was part of the explanation of the early expansions out of Africa, and the relatively modest increases in encephalization necessary to support even Oldowan toolmaking would have pushed past the primate grey ceiling, necessitating at least rudimentary forms of cooperative breeding. Edge-wear analysis of Oldowan tools suggests that they were used in butchery, the production of wood tools, cutting and scraping plants to eat, and perhaps for other purposes, such as cutting cordage [63]. By Acheulean times, about 780 ka, a rare waterlogged site suggests that humans were exploiting a wide variety of plant foods requiring cooking and other cultural processing techniques [64]. Hill *et al*.'s [11] hypothesis that critical elements of our complex cultural adaptation were already evolving around 2 Ma seems to be supported by subsequent work. The late date for the relatively small-brained *Homo naledi* fossils suggests that Lower Pleistocene brains and their cultures were viable long after more sophisticated cultures and larger brains evolved in other human lineages [65]. At the same time, life-history variation over the course of the Pleistocene was likely considerable, as is variation within modern human populations. In the Holocene, agricultural subsistence systems gradually came to dominate the Earth based upon the biotechnology of domestication [66]. The building of complex cultural adaptations takes many people and significant amounts of time, but nowhere near as much time as organic adaptations [67]. Social learning delivers powerful cultural tools into the hands of children and adolescents that raise their productivity much above that which they could achieve on their own. Without cultural tools, we could not pay the overhead costs of big brains and long life histories. The primate grey ceiling would be enforced on us too.

Thus, in principle, our large and costly brain might be explained by its ability to support more innate modules, better individual intelligence or cumulative culture. That so much of our subsistence is a product of sophisticated technology and social organization that is transmitted culturally suggests that cumulative culture is a major part of the answer.

## 3. Culture-specific life-history trade-offs

The four life-history characteristics enumerated by Kaplan *et al*. [2] could have been a product of adaptations to the cognitive and/or the cultural niche's demand for a big brain. However, there are other trade-offs that reflect specific demands that cultures and environments put on a life history that uses a big brain. Given that brains are organs of phenotypic flexibility, we might expect the brain's resources to be used differently by different individuals in the same environment, differently in different environments and differently in different cultures.

### (a) Large cultural networks

There is a fifth major life-history difference between humans and chimpanzees in addition to the four enumerated by Kaplan *et al*. [2]. Humans have very large social networks from which they can acquire culture compared with other apes and most other mammals [68]. When they learn socially, young chimpanzees learn important subsistence skills almost entirely from their mothers [69], as is the case in many others species. Learning from parents is also important in humans, but fathers not just mothers are important, and skills are gendered [70]. High paternity certainty in humans, possibly originally evolved to recruit fathers and fathers' relatives into allomaternal networks [71], would also have broadened social networks for purposes of social learning. The involvement of fathers and other males in child rearing of boys makes possible the male side of our gendered division of labour. Evidence suggests humans have a two-stage social learning system [72]. Young children imitate and are sometimes taught mainly by their parents, but peer play groups also seem to be important. Juveniles more actively seek out skilled non-parental adults to imitate.

The size of social networks people can use to access cultural variation is important because people can actively bias their acquisition of culture (and their teaching) [38]. Learners can often make informed choices about the utility of cultural variants. People tend to use or teach the best variant they know [73]. When it is difficult to judge which variant is best, learners can use rules of thumb like follow the majority or imitate the prestigious. Up to some limit, the more variation you can observe, the more likely you will learn a more valuable versus less valuable variant. At the population level, the rate of adaptive evolution can be quite rapid if networks are large and selective adoption and teaching are fairly effective. Classic examples include the rapid uptake of hybrid corn in the mid twentieth century [74].

Long-term quantitative studies of hunting and gathering groups demonstrate that they tend to live in fluid bands of 30–50 individuals that regularly exchange members, such that the whole ethnolinguistic tribe of a few hundred to a few thousand people participates in a common social network, a form of social structure that seems to be uniquely human [75]. Hadza and Ache males observe some 300 other men making tools in their lifetimes whereas chimpanzee males interact with only about 20 other males in a lifetime [68]. Such high interaction rates are probably necessary to sustain human cumulative cultures. Migliano *et al*. [76] studied social networks in Agta (Philippines) and BaYaka (Central Africa) and found them structured to make rapid cultural diffusion across families possible. Jordan [77] looked closely at the cultural evolution of different traditions in subsistence societies from three regions, two in western North America and one in western Siberia. He found that the evolution of a tradition was closely linked to the social networks of its makers. For example, boatbuilding on the Northwest Coast was a specialized craft and skilled boat-builders migrated fairly freely among communities, leading to very different patterns of boat form compared with house form. Buckley & Boudot [78] give the example of loom and weaving evolution in Southeast Asia and adjacent areas. Weaving is mostly passed on from mothers to daughters in long apprenticeships, leading to low rates of innovation. Loom design also evolves rather slowly. However, there is enough transfer of techniques within dialect communities to make historical relationships of loom designs and cloth very similar. Diffusion of looms and weaving designs between language communities is rare but not negligible in the long run. All the looms studied fit into a common phylogeny, suggesting a common cultural tradition on a subcontinental scale. Increases in the functional complexity of looms over time are the rule, but simplification in the interest of portability or other local concerns is not rare.

Sheer population size limits the scale of social networks and the level of social complexity that a societies can sustain [79,80]. Diamond [81] argued that this effect operates at the continental scale, and the impact of globalization after 1500 on the exchange of crops and technology is well known [82]. An interesting example is the economic and socio-political revolution set off in the rather isolated region of Highland New Guinea by the arrival of the American sweet potato about 300 years ago. Sweet potatoes provided a productive starchy staple that grew above the malaria belt, setting off a population explosion of people and pigs. A large exchange economy gradually evolved under the leadership of entrepreneurial ‘big-men' [83].

Increasing network sizes engenders trade-offs. Societies living at low population densities require costly investments in travel to maintain large social networks. In the Ju/hoansi (!Kung), a system of gift exchange links people in distant camps [84]. In the Western Desert of Australia, a section system requires young men to travel great distances in search of mates. Brides must be taken from a specific other section, and in the Desert, there are eight sections. In more densely populated regions, the number of sections tends to be fewer. Yengoyan [85] argued that in low density regions the elaborate section system forces costly travel just to maintain networks on a respectable scale. In the Upper Palaeolithic of Europe, population densities were quite low, but stylistically similar artefacts of the Gravettian culture are found from the Urals to the Atlantic and from the ice margins to the Mediterranean [86].

Increasing social network size also carries the risk of acquiring maladaptive ideas. Acquiring culture by vertical transmission is relatively safe in that parents and offspring are closely related genetically and tend live in the same ecological circumstances. Even so, parent–offspring conflict is a well-studied problem [87]. However, vertical transmission is very conservative, especially in the face of spatial and temporal variation and the existence of potentially very useful ideas in other lineages. We can expect that genetic and cultural evolution have favoured the evolution of social learning strategies that manage social networks in order to minimize the impact of this trade-off [88–90]. The literature on the diffusion of innovations has classic empirical examples of how individuals strategize their information acquisition [91]. Take ethnicity as an example [92,93]. In theory, symbolic markers of group identity such as dialect, dress or ritual can evolve to limit the acquisition of maladaptive cultural variants. Neighbours who live in different ecological circumstances could be a source of subsistence ideas unsuited to one's own environment. Different social systems tend to solve coordination problems in different ways, and imitating neighbours could mis-coordinate you with your group mates. Neighbours may care less about your welfare than group mates and might promote the spread of ideas in their interest but not in yours. The diffusion of innovations literature is rich in examples where the markers of ethnicity, class, gender, political affiliation and the like reduce the chances of innovations spreading. Young children have a marked bias towards imitating people like themselves. Dialect seems to be an especially important cue [94]. At the same time, people are strongly attracted to acquiring innovations that work well. For example, stone tool makers rapidly see the advantages of steel tools and abandon their traditional stone if they have a ready supply of steel replacements [95].

Human social networks are poly-functional. A person's information network, network of relatives, network of economic partners and network of acquaintances have different costs and payoffs yet they tend to heavily overlap. For example, Thornhill & Fincher [96] defend the hypothesis that humans use markers like ethnicity to avoid contagious microbial infections, not contagious bad ideas. Since networks from which one might acquire bad ideas and bad infections broadly overlap, the two hypotheses make similar empirical predictions. Hence attributing properties of social networks to trade-offs related to information acquisition or any other function is difficult. Advances in techniques to study multiplex networks might make progress on this front possible [97].

### (b) Investment in teaching and social learning versus individual learning

Do humans show any signs of being adapted to social as opposed to individual learning? At least at the margin of time these activities trade-off against one another even if both capacities extensively share cognitive resources. A now voluminous literature documents that children are adept at social learning even as compared with other apes [98,99]. This comparative work has been able to dissect the proximate reasons for our advantage. For example, children use language to assist other children to acquire a solution to a hard task, something other primates cannot do [100]. Children avidly learn social norms, an apparent adaptation to our rule-bound social systems [101]. Children readily learn concepts like ‘oxygen' and ‘god' even when they cannot use their own evidence to support such concepts [102]. On the teaching side, children are sensitive to ostensive clues, like pointing, offered by adults to assist in the child's learning and children also point to solicit things like the names of objects from adults [103]. Csibra & Gergely [104] argue that natural pedagogy is part of the human social learning adaptation. See also [72]. Learning and social learning in hunter–gatherers has recently been reviewed by contributors to [105]. Humans certainly seem adapted to employ social learning.

## 4. Conclusion

The evidence we have reviewed suggests that the long, slow human life history coevolved with our large brain. Brains are substantially organs of phenotypic flexibility, and brain size increased in many mammalian lineages in the Cenozoic, with humans holding down the upper tail of the distribution of brain size relative to body weight. This evolution seems to have been driven by increasingly variable environments. The question is how do humans, and by extension other large-brained creatures, pay the high overhead costs of large brains? The general answer seems to be learning and other forms of individual creativity plus social learning. The extraordinarily large modern human brain depends upon high skilled food acquisition strategies that make nutrient-dense foods available by exploiting a great variety of locally available food resources. Cooperative breeding, especially the heavy involvement of men in helping provisioning of mothers with dependent offspring, requires institutions of marriage and kinship. Culturally transmitted subsistence skills and techniques and culturally transmitted social institutions make possible a life history that is simultaneously slower but capable of higher completed family size than in other apes.

## Data accessibility

This article has no additional data.
